# Muscarinic Receptors and Alzheimer’s Disease: New Perspectives and Mechanisms

**DOI:** 10.3390/cimb46070407

**Published:** 2024-07-02

**Authors:** Martina Monaco, Hanna Trebesova, Massimo Grilli

**Affiliations:** 1 Department of Pharmacy, University of Genoa, Viale Cembrano 4, 16148 Genoa, Italy; martina.monaco@edu.unige.it (M.M.); hanna.trebesova@unige.it (H.T.); 2 Inter-University Center for the Promotion of the 3Rs Principles in Teaching & Research (Centro 3R), 16148 Genoa, Italy

**Keywords:** Alzheimer’s disease, muscarinic receptors, cholinergic transmission, neuroinflammation, innovative therapy

## Abstract

Alzheimer’s disease (AD) is one of the most prevalent neurodegenerative diseases on a global scale. Historically, this pathology has been linked to cholinergic transmission, and despite the scarcity of effective therapies, numerous alternative processes and targets have been proposed as potential avenues for comprehending this complex illness. Nevertheless, the fundamental pathophysiological mechanisms underpinning AD remain largely enigmatic, with a growing body of evidence advocating for the significance of muscarinic receptors in modulating the brain’s capacity to adapt and generate new memories. This review summarizes the current state of the art in the field of muscarinic receptors’ involvement in AD. A specific key factor was the relationship between comorbidity and the emergence of new mechanisms.

## 1. Introduction

### 1.1. Alzheimer’s Disease Impact

In 2022, according to the American Journal of Managed Care, the total cost of Alzheimer’s disease (AD) and associated dementia treatment was USD 321 billion and is expected to increase to reach USD 1 trillion in 2050. Almost half of these costs are due to medical care, but take into account medical assistance and care communities [1]. These numbers come from the USA, but then, if considered globally, WHO reports that AD is the most common form of dementia, contributing to 60–70% of cases. The worldwide impact of dementia was already USD 1.3 trillion in 2019 (World Health Organization, WHO, Dementia (who.int)).

### 1.2. Alzheimer’s Disease Pathophysiology

AD is a progressive neurodegenerative disease that, to date, does not have a specific cure. Multiple pathological mechanisms have been proposed: amyloid beta (Aβ) accumulation, neurofibrillary tangles formation, and tau hyperphosphorylation [2]. Although the amyloid cascade hypothesis is currently the most studied theory for the development of AD, there are some more recent hypotheses, such as neuroinflammation, oxidative stress, and mitochondrial dysfunction [3]. Mechanisms, such as the gut–brain axis involvement, vascular dysfunction, and autophagy, also seem to be implicated in AD [4]. The overall picture gives us a devastating, probably multifactorial pathology that we intercept too late.

It is known that neuroinflammation plays a crucial role in the development and progression of Alzheimer’s disease [5,6]. For example, the accumulation of Aβ and hyperphosphorylated tau protein aggregates in AD triggers the activation of astrocytes and microglia, releasing pro-inflammatory cytokines and reactive oxygen and nitrogen species, which contribute to neuroinflammation [6]. This inflammatory response can have a dual function, with both protective and detrimental effects on AD progression [7]. Indeed, inflammatory cytokines, such as interleukin 1-β (IL-1β), interleukin-6 (IL-6), and tumor necrosis factor-alpha (TNF-α), are increased in AD and mild cognitive impairment (MCI) patients. High levels of IL-6 have been associated with an increased risk of cognitive decline [8]. Exaggerated release of reactive oxygen species within the brain may lead to a neuroinflammatory type of AD. Activation of microglia through these reactive compounds can induce the release of multiple pro-inflammatory cascades and general brain atrophy [9]. Dysbiosis of the gut microbiota can prime neuroinflammation and the deposition of Aβ plaques, characteristic of this pathology [10]. AD has been linked to gut microbiota alterations, with studies showing the presence of gut dysbiosis in AD patients [11]. The gut microbiota can affect the brain and behavior of AD patients, including their cognitive function [12]. From a mechanistic point of view, a reduction in short-chain fatty acid-producing strains may be involved in the pathology of AD [13,14]. Accordingly, dietary supplementation with prebiotics or fatty acids improved both histopathological and cognitive aspects in animal models of AD [15]. 

Historically, a cholinergic hypofunction and a degeneration of cholinergic neurons seem to be associated with this pathology [16]. The loss of cholinergic elements in the brain, a characteristic feature of AD, may be due to the toxic interaction of protein tau with muscarinic acetylcholine receptors (mAChRs) [17]. Among the receptors of the cholinergic system, the mAChRs participate in acetylcholine (ACh)-induced neurotransmission and have been implicated in AD [18]. Nevertheless, nicotinic receptors are also involved in AD [19,20,21,22,23]. It is of interest to note that these receptors can interact with mAChRs, giving rise to functional crosstalk phenomena that can be influenced by the action of Aβ [24,25]. In addition, it is well known that a disruption in cholinergic neurotransmission leads to cognitive impairments and behavioral alterations [26,27]. 

### 1.3. Early Hallmarks of AD

Converging hypotheses sustain that a delayed diagnosis could limit the discovery of effective therapies [28,29,30]. In agreement, it has become critical to identify early symptoms [31,32]. Before the evidence of clinical symptoms, several neurodegenerative diseases, including AD, could be anticipated by sleep alterations [33,34]. Hence, maintaining sleep might be effective in slowing AD appearance or progression. One potential therapeutic target for improving sleep is fatty acid amide hydrolase, as suggested in a study by Martin et al. [34]. In their study, an acute inhibition with PF3845, a selective inhibitor of fatty acid amide hydrolase, improved sleep behaviors in male and female transgenic Tau P301S mice (model of Tauopathy and AD [35]). Unfortunately, under chronic conditions, the treatment was not able to counteract progressive sleep loss, neuroinflammation, and cognitive decline [34]. However, sleep disruption and circadian rhythm alteration are also present in patients with frontotemporal dementia [33]. In a study conducted by Filardi, patients underwent actigraphy to monitor their sleep. Although the sleep duration increased in these patients, the quality of sleep decreased. Furthermore, the study has found that sleep duration is associated with reduced cortical thickness in frontal regions, as calculated by magnetic resonance imaging [33]. AD patients present diffuse brain atrophy regions, but some regions show an increase in size. Additionally, recent work has suggested neural facilitation in areas that are anticorrelated with atrophied regions in frontotemporal dementia [36]. 

Recent investigations have demonstrated that AD plays a pivotal role in the disruption of lipid metabolism, which is a significant contributing factor in the development of the disease [37]. The overexpression of the familial amyloid precursor protein (APP) calls for particular consideration, particularly in light of the advances in translational molecular imaging. These developments have made it feasible to probe cholesterol metabolism in the living human brain using positron emission tomography. This presents an appealing prerequisite for future clinical trials aimed at targeting the brain cholesterol machinery in patients with AD [38]. 

### 1.4. AD and Diabetes

The difficulty in finding an effective therapy for AD led us to evaluate its relationship with other diseases [39,40]. In particular, the association with diabetes seemed rational [41,42]. Therefore, understanding the link between AD and diabetes could lead to new strategies for treating both diseases together. AD and diabetes share several metabolic similarities. These include mitochondrial dysfunction and cellular lipotoxicity. Mitochondria produce less energy when they are dysfunctional, leading to oxidative stress and inflammation—factors that contribute to both diseases. Cellular lipotoxicity, the accumulation of harmful lipids in cells, can damage mitochondria, disrupt insulin signaling and promote inflammation. This increases the risk of AD and type 2 diabetes. Insulin has been shown to both clear Aβ in the brain and impact tau protein phosphorylation while also improving synaptic activity and neuron plasticity in both human and animal studies [43,44]. These metabolic connections between AD and diabetes open new therapeutic possibilities: some antidiabetic drugs, such as metformin, may have beneficial effects on cognition and reduce the risk of AD in diabetic patients [45]. Recent studies have provided promising evidence for using glucagon-like peptide-1 (GLP-1) receptor agonists to treat both diabetes and AD. These findings suggest that it may be possible to reduce the risk of developing dementia by using GLP-1 receptor agonists to target the underlying pathophysiological mechanisms associated with both diseases. This provides a novel approach that could potentially result in the development of a common therapy for both diabetes and AD, with significant implications for managing both conditions. 

### 1.5. Treating Alzheimer’s Disease: Managing Symptoms and Exploring New Targets

The current state of the art in AD therapies involves the use of acetylcholinesterase (AChE) inhibitors to enhance cholinergic neurotransmission and improve cognitive and behavioral symptoms [46]. These inhibitors, such as donepezil and galantamine, have shown moderate clinical benefits in treating AD [21]. The “cholinergic hypothesis of AD” assumes that memory and learning deficits result from the loss of cholinergic innervation to the cortex and hippocampus from nuclei in the basal forebrain. It was demonstrated that reduced ACh synthesis and degeneration of cholinergic innervation are principal contributors to AD [47]. Thus, one of the most pursued therapeutical strategies is the inhibition of AChE to increase ACh levels or aimed at directly stimulating cholinergic receptors. However, these treatments manage only a part of the symptoms and are not devoid of side effects, especially gastrointestinal [48]. Additionally, the use of N-methyl-D-aspartate (NMDA) receptor antagonists, like memantine, has been found to have a clinical effect on behavioral symptoms and is often combined with AChE inhibitors to enhance their efficacy [17]. ACh is assumed to be active in memory formation by potentiating NMDA receptor currents by M_1_ mAChRs [49]. Accordingly, mAChRs have been identified as potential targets for new drugs in AD therapy [50]. The primary mAChRs, M_1_R, and the α7 nicotinic ACh receptor subtype have been the focus of recent experimental targets for mAChR agonist strategies [51]. The development of more selective M_1_R compounds, including positive allosteric modulators (PAMs), is being explored to improve cognitive function and potentially modify the progression of AD [52,53]. PAMs for the M_1_R mAChRs have shown promise in preclinical models for the treatment of AD [48]. These PAMs can enhance the binding and efficacy of ACh, improving impaired cholinergic transmission associated with AD [54]. A significant advance in the treatment of AD is the development of monoclonal anti-Aβ antibodies, although their real effectiveness, established in clinical trials, is still controversial [55,56,57]. It is established that even those compounds were unable to block the disease, let alone reverse the cognitive decline of AD patients. Their main function appears to be to slow down the rate of decline in cognitive and everyday functions. Interestingly, a recent randomized clinical trial, DIAN-TU-001, reports the beneficial impact of fibrillar Aβ reduction on fluid markers of synaptic dysfunction and neuroinflammation induced by gantenerumab and solanezumab [58]. Therefore, these anti-Aβ antibodies could be incorporated into AD drug therapy in conjunction with other drugs, lifestyle modifications, and supportive care for the patient and family members [58,59].

## 2. Muscarinic Receptors’ Involvement in Pathological Conditions

**Figure 1:**
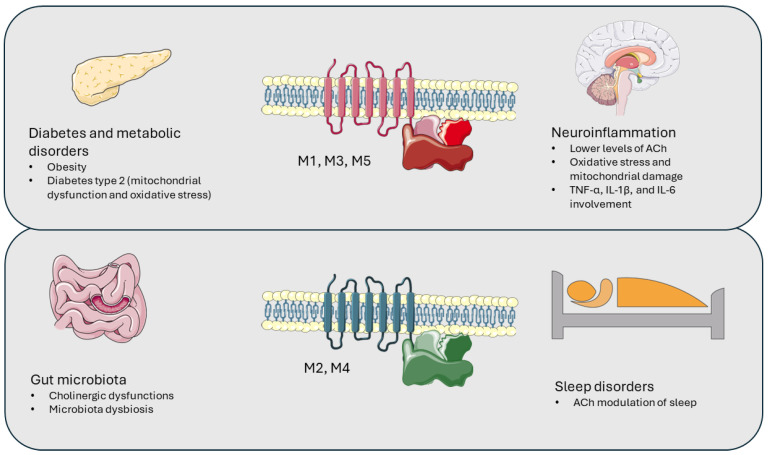
Contribution and involvement of muscarinic receptors in some pathological conditions. The figure was partly generated using Servier Medical Art, provided by Servier, licensed under a Creative Commons Attribution 3.0 unported license (https://smart.servier.com/, accessed on 27 May 2024).

Dysfunctions of mAChRs are present in AD, Parkinson’s disease, and schizophrenia [60]. The muscarinic receptor consists of the M_1_-M_5_ muscarinic receptors. Each subtype has defined functions in the nervous system and mediates the actions of ACh. All mAChRs are G-protein coupled receptors (GPCRs) [61]. M_1_Rs are widely distributed in the cerebral cortex, hippocampus, and basal ganglia. The M_1_R subtype is considered an important potential therapeutic target for AD [16]. M_2_R regulates neurotransmitter release and ACh itself. M_3_Rs are poorly represented in the brain, while M_4_Rs are found in the striatum, hippocampus, and cortex. They are implicated in the regulation of dopamine release and have been associated with conditions such as schizophrenia and Parkinson’s disease. M_5_R is also involved in regulating dopamine release and is distributed in the same brain regions [61]. Historically, the involvement of mAChRs in AD has been proposed. However, it is now crucial to evaluate the involvement of these receptors in the pathological mechanisms that have been associated with AD (Figure 1, Table 1).

### 2.1. Muscarinic Receptors and Neuroinflammation

Neuroinflammation is a complex process characterized by the activation of the brain’s immune cells in response to numerous factors such as injury, infection, or neurodegenerative conditions. This immune response can contribute to the progression of neurodegenerative diseases. The scientific evidence indicates that mAChRs are also involved in neuroinflammation and may regulate phenomena associated with neurodegenerative diseases [89]. Gatta et al. state that there is evidence that the levels of ACh are lower in multiple sclerosis patients: this suggests that a deficiency in the cholinergic system may be correlated with neuroinflammation, typical of neurodegenerative diseases [90]. According to research conducted on blood, serum, and cerebrospinal fluid from multiple sclerosis (MS) patients as well as those conducted on animal models of the disease, cholinergic changes may be a factor in the dysregulated inflammatory processes associated with multiple sclerosis [91]. Interestingly, Oxotremorine, a non-selective mAChRs agonist, prevents DNA fragmentation caused by the Aβ_1–42_ peptide and improves cell survival in SH-SY5Y neuroblastoma cells [92]. The same treatment inhibited oxidative stress and mitochondrial morphological/functional damage linked to exposure to Aβ_1–42_ cells. Another study performed on AD patients also determined that decreased levels of ACh and increased neuroinflammation were associated with cognitive decline, suggesting that the use of anticholinesterase drugs could lead to an improvement in the clinical condition of these patients, especially if an early diagnosis can be made [93]. Furthermore, mAChRs are also present in the microglia. Both endogenous and external inflammatory signals are detected by microglia, which then coordinates the ensuing neuroinflammation [94].

### 2.2. Muscarinic Receptors and Gut Microbiota

In 2020, Teratani and colleagues published a groundbreaking article revealing the presence of a liver–brain–gut neural arc that fine-tunes immune responses in the intestine. This pathway involves mAChRs and predisposes the gut to inflammation, suggesting a potential role in the development of neural diseases [95]. A study by Wu et al. explored the potential of pyridostigmine (PYR), a cholinesterase inhibitor that boosts vagal activity, to regulate the disrupted gut microbiota in diabetic mice. Their findings revealed that PYR effectively alleviated gut microbiota dysbiosis [96], suggesting a connection between the cholinergic system and the gut microbiota. Since dysbiosis of the gut microbiota seems to be a prodrome to the onset of AD, as well as cholinergic dysfunction, it could be extremely useful to use drugs that act on the cholinergic system to treat intestinal disorders as well.

### 2.3. Muscarinic Receptors and Sleep Disorders

ACh plays a crucial role in the regulation of arousal and sleep, and the vigilance state is correlated with considerable changes in its release in different regions of the brain [97,98]. M_2_Rs and M_4_Rs seem to be involved in the modulation of sleep during homeostatic challenge. Accordingly, M_2_Rs are reduced in rats who are sleep-deprived [99]. It has long been clear that anticholinesterase drugs, such as donepezil, already used in AD patients, can also contribute to the treatment of sleep disorders. This happens because by increasing the neurotransmitter ACh in the brain, the latter, by binding to mAChRs, increases rapid eye movement (REM) sleep [100,101].

### 2.4. Muscarinic Receptors and Metabolic Disorders

The significance of muscarinic receptors in the context of metabolic disorders such as obesity and subsequent diabetes is of considerable importance. The research highlights the significance of mAChRs in regulating energy homeostasis and metabolic health, suggesting their potential as therapeutic targets for managing obesity and type 2 diabetes [102]. The effect of cevimeline, an M_3_R agonist, on olanzapine-induced metabolic disorders in rats has been investigated. Co-treatment of cevimeline with olanzapine significantly attenuated olanzapine-induced weight gain, dyslipidemia, and impaired glucose metabolism by modulating the hepatic M_3_R-AMPKα pathway and improving AKT-GSK3β signaling. This highlights the importance of targeting M_3_R-related pathways to manage metabolic disturbances [103].

## 3. Muscarinic Receptors as a Pharmacological Target

### 3.1. M_1_: Conceivable AD Target

Activation of the M_1_Rs has shown potential as a disease-modifying target in several neurodegenerative diseases. In particular, M_1_ mAChRs have been shown to play a role in cognitive dysfunctions associated with AD [62]. Aβ has been found to dysregulate mAChRs activity [64]. Aβ oligomers, which accumulate in the brains of the patients, disrupt synaptic plasticity and dendritic loss through interactions with NMDA receptors (NMDAR) and metabotropic glutamate receptor 5 (mGluR5) [18]. The dysregulation of mAChRs by Aβ-mediated activation of mGluR5 negatively affects mAChRs function [104]. M_1_Rs deletion in cortical neurons affects mitochondrial function and ultrastructure; this produces mitochondrial pathophysiological deficits in AD [67]. In the context of M_1_Rs as a therapeutic target, a recent study on C57BL/6 mice by Huff and colleagues suggests that M_1_R activation promotes the updating of object location in memory tasks [63]. Mice were evaluated in object-location and object-updated-location tasks with or without scopolamine, non-selective mAChR antagonist, and MK-801, non-selective NMDAR antagonist. The study concluded that scopolamine was able to prevent novelty-induced destabilization and memory update in localization tasks. Likewise, MK-801 impairs object location memory reconsolidation in mice [63]. MK-801 seems to provoke deficits in social memory alongside the prairie voles model of schizophrenia [105]. This finding strengthens the potential of M_1_R agonists as therapeutic agents for AD, as they may not only improve cognitive function but also enhance the brain’s ability to adapt and form new memories. Recently, an ultrastructural observation consistent with electrophysiological studies has demonstrated M_1_Rs and NMDARs co-localization in hippocampal dendrites [49], and activation of M_1_Rs has been shown to play a crucial role in mnemonic functions in the anterior basolateral nucleus of the amygdala. Consequently, targeting M_1_Rs by PAMs could ameliorate memory impairments in neurodegenerative disorders such as AD [49,106]. On the other hand, even sensory stimuli could be implicated in memory formation. Mishra and colleagues have investigated the primary somatosensory cortex in mice and its influence on the animal’s sensitivity to detect vibrotactile stimuli [26].

Among new therapeutic strategies, broad neuroprotection rather than direct Aβ or hyperphosphorylated tau targeting seems to be encouraging. In particular, a mixed action on M_1_Rand σ_1_ chaperone proteins of the endoplasmic reticulum (ER) has been investigated in recent years [65]. In the presence of chronic ER stress, σ_1_ chaperone protein activates the Ca^2+^-dependent intracellular cascade, increasing or modifying σ_1_ protein activation, using agonist, therefore exerting a pharmacological action on Ca^2+^ homeostasis and simultaneously activating neuroprotective pathways [65]. This double effect was found in ligands of the ANAVEX series [35,65,66]. One of the promising compounds, ANAVEX2-73, also known as blarcamesine, was able to block Aβ_25–35_-induced Recognition Memory Deficits to prevent tau hyperphosphorylation and Aβ_1–42_ seeding in Aβ_25–35_-injected mice [65]. ANAVEX2-73 has shown good results in a mouse model of Rett syndrome, a severe neurodevelopmental disease that is often associated with mutations in the transcriptional regulator *MECP2*, improving motor coordination and balance, sensory, acoustic and visual responses, showing a good safety profile due to a low dose (10 µg/kg) that minimizes the possibility of peripheral cholinergic side effects [66]. ANAVEX2-73 has passed the phase 3 study for AD and Rett syndrome; additional studies for the treatment of PD and related dementia are planned for this compound.

Another interesting candidate is ANAVEX3-71, known as AF107B, which is more focused on AD and frontotemporal dementia; at the moment, this compound has finished phase 1, and phases 2 and 3 are planned [35,68]. This compound was tested on 3xTg-AD mice and McGill-R-Thy1-APP, attenuating cognitive deficits and mediating APP metabolism; no side effects were observed at a maximum dose of 50 mg/kg. Clinical studies have evaluated the pharmacokinetic profile (PK) of ANAVEX3-71 and its active metabolite M8 in health candidates. The studies concluded that the PK was linear, dose-proportional, and time-invariant. Additionally, food does not affect the PK of both compounds and their metabolites [69]. Assessing the effect of both on electrocardiogram (ECG) parameters in healthy participants did not result in clinically relevant alterations to the cardio-dynamic profile [70].

### 3.2. M_2_: Complex Non-Selective Target

The M_2_R is moderately abundant throughout the brain, and it is present in particular on cholinergic neurons and nonpyramidal neurons in the cortex and hippocampus. Like all mAChRs, it plays a role in modulating neurotransmitter release, synaptic transmission, and cognitive function [71]. A study highlighted that when specific subtypes of M_1_Rs and M_2_Rs are activated in tissue slices, it affects the processing of APP [71]. The activation of M_2_R suppresses the non-amyloidogenic processing pathway of APP, while the activated M_1_R leads to an increase in the expression of BACE1 (beta-site APP cleaving enzyme 1), an enzyme involved in amyloid plaque formation. These findings suggest that the cholinergic system plays a significant role in APP processing and in the development of AD. In this context, bi-pharmacophoric inhibitors of the cholinesterase with affinity to the M_1_Rs and M_2_Rs were tested, and one of the compounds resulted in a good candidate for the development of an anti-AD drug [72]. Notably, there is a selective decrease in M_2_R in AD-affected brain regions containing senile plaques [107]. Another study highlighted the reduction in M_2_R density detected in specific brain regions of AD patients [107]. Subgroup analyses revealed differential alterations in neurochemical variables among AD patients exhibiting various behavioral symptoms, with an increase in M_2_R density observed in select symptom groups. Notably, delusions predicted variability in M_2_R density in one brain region, while hallucinations were positively associated with M_2_R density in another region [73]. Although recent studies have focused more on other mAChR subtypes, several compounds selective for M_2_R have been highlighted in the past. Early compounds targeting M_2_R lacked the desired combination of M_2_R affinity, subtype selectivity, and central nervous system (CNS) activity. SCH57790 showed promising M_2_R binding affinity and selectivity, leading to further development of other compounds. Because M_2_R activation inhibits ACh release in brain regions that are critical for learning and memory processes, M_2_R antagonists have been proposed as a potential treatment for AD, as they can enhance cognition by facilitating ACh release in the brain [74].

Both sporadic and familial AD display cholinergic and lipid dysregulations [108]. In postmortem brain of AD patients, the distribution of phospholipids/sphingolipids and the activity of cannabinoid 1 (CB1), sphingosine 1-phosphate 1 (S1P1), and muscarinic M_2_/M_4_ receptors in the frontal cortex were investigated through MALDI-mass spectrometry imaging [109,110]. Compared to patients with MCI and not cognitively impaired subjects, AD patients have presented a downregulation of phosphatidylinositol levels in white matter compared to controls. On the other hand, M_2_/M_4_Rs activation was decreased in AD patients [110].

In the case of AChE inhibitors used in patients with mild to moderate dementia, it is necessary to consider possible side effects. In particular, peripheral M_2_Rs could cause bradycardia, conduction abnormalities, and hypotension [111].

### 3.3. M_3_: Involvement in Mood Disorders and Sleep Functions

Similarly to M_1_R and M_5_R, the M_3_R is coupled to Gq-11, stimulating phospholipase C and inositol phosphate, thereby mediating an excitatory effect through intracellular calcium influx [112]. M3Rs are poorly expressed in the CNS [113]. To date, only a limited number of studies have explored changes in M_3_R expression in the frontal cortex in the context of mood disorders, which could also be linked to AD [75]. An analogous consideration should be made for M_2_R and M_3_R in terms of side effects [114]. Lu 25-109 has failed to produce a significant difference in cognitive or behavioral symptoms of AD [76], probably because it also acts as an M_2_/M_3_Rs antagonist [77].

A recent study has shown that M_1_R and M_3_R encoded by CHRM_1_ and CHRM_3_ genes, respectively, are involved in sleep functions [78]. Sanfilippo and colleagues have demonstrated that the expression levels of CHRM_1_ and CHRM_3_ were significantly reduced in AD brains, compared with those of age and sex-matched non-demented healthy control brains. Moreover, these associations were found to be modulated by sex. Accordingly, males expressed higher levels of CHRM_1_ and CHRM_3_ than females in the temporal and occipital regions of healthy subjects. In AD patients, males exhibited higher levels of CHRM_1_ and CHRM_3_ in the temporal and frontal regions, respectively, than females [78].

Furthermore, another study has investigated the role of M_1_R and M_3_R in learning and plasticity at the hippocampus level [80]. In particular, the study has focused on the mossy fibers (MF)–CA3 pyramidal cell synapse obtained from hippocampal slices of double knockout mice. Interestingly, it was found that long-term plasticity was affected by reduced excitatory synaptic drive onto CA3 pyramidal cells rather than short-term plasticity. Previous studies have demonstrated similar effects on behavioral flexibility, working memory, and hippocampal plasticity in M_2_R-knockout mice [81]. Brain TNF-α, IL-β, and IL-6 mRNA expression were induced by acute M_3_ activation. Nevertheless, this “pro-inflammatory” effect was eliminated upon repeated muscarinic activation, showing that repeated activation of M_3_Rs in microglia contributes to the development of microglial tolerance. As the researchers themselves point out, further studies are needed, but this finding gives hope for the development of new selective therapies.

### 3.4. M_4_: Promising AD Target

Nevertheless, the M_1_R is well documented; in the literature, several studies have investigated the effects of M_1_/M_4_R-acting compounds. Conversely, M_4_R modulation has been less extensively studied [60,85]. The M_4_R is primarily expressed in the brain, specifically in regions such as the striatum and the hippocampus. M_4_R is present in the cortex, expressed and found presynaptically as autoreceptors in glutamatergic synapses on neurons, and is particularly enriched presynaptically within the substantia nigra [82]. Moreover, structure-based drug discovery enables the development of orthosteric agonists that are highly selective for mAChRs, including the M_1_ and M_4_ subtypes, which play a pivotal role in cognitive and behavioral modulation [82]. In the study conducted by Wang and colleagues, several ligands were investigated for their activity on M_4_Rs [83]. A promising compound-110 has demonstrated allosteric agonist binding with M_4_, resulting in antipsychotic activity in a schizophrenia mouse model. Additionally, it has been observed to reverse MK-801-induced hyperlocomotion without inducing catalepsy in mice [83]. Given that cholinergic neurons degenerate with the progression of the disease, it may be more advantageous to target postsynaptic M_4_Rs, which are relatively preserved in AD, with orthosteric agonists [82].

This receptor is co-expressed in a subpopulation of dopamine-1 receptor (D1R)-expressing medium spiny projection neurons in the striatum, where it plays a role in modulating striatal dopamine release and dopamine-related behaviors [82]. Anomalies in dopaminergic transmission can affect synaptic plasticity, motor activities, and cognitive behavior, which are essential aspects of AD. Moreover, the existing evidence strongly supports the occurrence of functional interactions between M4Rs and DR at different levels [115,116,117]. M_4_Rs exert inhibitory control over D1R-mediated locomotor stimulation, likely at the level of striatal projection neurons where both receptors are highly co-expressed [115]. M_4_R is a promising therapeutic target for the treatment of movement disorders and additionally acts as a disease modifier due to its cholinergic activity [84]. The role of M_4_Rs in dopaminergic signaling, which is involved in movement, is relevant in the context of both AD and Parkinson’s disease [60,84].

M_4_R agonists have demonstrated positive effects on psychiatric and cognitive symptoms in patients with schizophrenia and related psychotic disorders, while reductions in M_4_R activation have been associated with hippocampal deficits thought to underlie the amnestic features of AD [82]. Xanomeline, a multitargeting drug with muscarinic and serotoninergic activities [118], has the potential to enhance wakefulness and arousal in aging, MCI, and AD patient populations. This is suggested by its ability to reverse wake fragmentation and disruptions in arousal in nonpathologically aged mice [85]. The activation of M_4_R in rodents’ brains using a compound called PT-3763 reduced the amount of input received by CA1 and CA3 pyramidal neurons in a dose-dependent manner when assessed ex vivo. Furthermore, the administration of this compound throughout the body or directly into specific brain areas (in vivo) reduced the activity of CA1 pyramidal neurons in a dose-dependent manner. When PT-3763 was systemically administered, it resulted in a reduction in the release of the neurotransmitter glutamate from CA3 pyramidal neurons. Treatment with this compound also improved the performance of rodents in the Morris water maze [86]. Novel selective M_4_R PAMs have been developed, demonstrating efficacy in preclinical models predictive of antipsychotic and pro-cognitive effects. Selective M_4_R PAMs have shown potential for managing neuropsychiatric symptoms associated with AD, such as agitation and aggression, with improved therapeutic margins for cholinergic adverse effects compared to less selective muscarinic receptor agonists [119]. To quantify M_4_R engagement in the brain, positron emission tomography (PET) tracers such as [^11^C]MK-6884 and [^11^C]PF06885190 have been developed, which have aided in the development of new drugs and the conduct of clinical trials [119,120]. The PET ligand [^11^C]MK-6884 has demonstrated selectivity for the muscarinic M_4_R subtype over other subtypes and exhibits high brain uptake. It can indicate changes in M_4_R density and may provide crucial insights into the diagnosis of neurodegenerative diseases, including AD. A study evaluated [^11^C]MK-6884 in AD, MCI, and normal healthy volunteer samples. A reduction in muscarinic M_4_R density was observed in AD tissues in comparison to normal healthy volunteer samples [121].

### 3.5. M_5_: Relevant Role in Memory Impairment in AD

The M_5_R is a receptor that is coupled to Gq/11 proteins. It has been successfully cloned in humans and exhibits structural similarities with the M_3_R. This mAChR is expressed in various brain regions, including the hippocampus, hypothalamus, substantia nigra, and ventral tegmental area [122]. Araya and colleagues generated M_5_R knockout mice to elucidate the receptor’s role in cerebral ischemia. These rodents exhibited reduced cerebral blood flow in the cortex, hippocampus, basal ganglia, and thalamus. In vivo experiments were conducted, and magnetic resonance angiography revealed that the decrease in blood flow to hippocampal pyramidal neurons resulted in significant neuronal atrophy and memory impairment in the Novel Object Recognition protocol. This suggests the potential use of M_5_R as a target to be reached for the treatment of memory impairment in AD patients [87]. Wieronska and colleagues have found that activators of M_5_R, as well as M_1_ and M_4_ mAChRs, effectively counteract several aspects of MK-801-induced memory impairment in the Morris Water Maze test. This highlights the significance of these receptors in a multitude of cognitive functions and cyclic guanosine monophosphate (cGMP)-dependent processes [88]. The expression of M_5_Rs in these cells was confirmed via reverse transcription polymerase chain reaction and immunoblotting. The use of short interfering RNA (siRNA) silencing demonstrated that the downregulation of M_5_Rs resulted in a significant reduction in the Ca^2+^ response to ACh, thereby confirming the role of this receptor in mediating ACh-induced Ca^2+^ signals in hCMEC/D3 cells [123].

## 4. Supporting Data

The review is supported by the literature that was gathered using the PubMed, Scopus, and Google Scholar databases, as well as from papers cited within the initial articles retrieved. Search terms included combinations of the following words: Alzheimer’s disease, neuroinflammation, metabolic disorders and AD, gut–brain axis, microbiota and AD, muscarinic receptors and sleep disorders, new pharmacological targets for muscarinic receptors, and similar combinations.

## 5. Conclusions

In conclusion, the hypothesis that mAChRs play a role in AD remains a recurrent topic of interest (Table 1). The notion that this theory has spanned decades of research and resurfaces periodically without foundation seems implausible. The muscarinic component appears to be most involved in aspects related to memory and the cognitive sphere. It can be postulated that alterations in these receptors may also contribute to the relationship between AD and certain emerging comorbidities. It should be noted that this review has some limitations, such as the lack of precise analysis of side effects. However, this aspect is partially due to the need for additional studies to clarify the effectiveness of new therapeutic approaches. Accordingly, we have indicated side effects for compounds already tested in clinical trials. Finally, this review is part of a comprehensive existing literature that presents a multitude of high-level works with the objective of summarising the latest evidence in the context of comorbidity with AD.

## Figures and Tables

**Table 1:** The dysregulation and potential positive effects of action on mAChRs.

Subtype	Negative Effects	Reference	Positive Effects	Reference
**M_1_**	Cognitive dysfunction	[62]	Improvement in object location in memory tasks	[63]
	mAChRs dysregulations	[64]	σ1 chaperone collaboration, Ca^2+^ homeostasis (ANAVEX2-73)	[35,65,66]
	Mitochondrial function alterations	[67]	Modulation of APP metabolism (ANAVEX3-71)	[68,69,70]
**M_2_**	Interfere with APP processing	[71]	Bipharmacophoric inhibition of cholinesterase (10-C10)	[72]
	Hallucination and behavioral symptoms	[73]	Enhancement of cognition by facilitating ACh release (SCH577790)	[74]
**M_3_**	Mood disorders	[75]	Cognitive and behavioral improvement (LU 25-109)	[76,77]
	Sleep dysfunctions	[78]	PAM activity (N-pyrimidyl/pyridyl-2-thiazolamine)	[79]
	Reduced excitatory synaptic drive onto CA3 pyramidal neurons	[80,81]		
**M_4_**	Locomotor alterations	[82]	Cognitive and behavioral modulation (compound-110)	[82,83]
			Treatment of movement disorders	[84]
			mAChR agonist (Xanomeline)	[85]
			Reduction in glutamate release from CA1 and CA3 pyramidal neurons (PT-3763)	[86]
**M_5_**	Reduced blood flow in the hippocampus results in neuronal atrophy and memory impairments	[87]	M_5_ activation (VU0357017, VU0152100, VU0238429)	[88]

## Data Availability

No new data were created; all information was gathered from already published papers.
